# Noise-Assisted Concurrent Multipath Traffic Distribution in Ad Hoc Networks

**DOI:** 10.1155/2013/543718

**Published:** 2013-11-10

**Authors:** Narun Asvarujanon, Kenji Leibnitz, Naoki Wakamiya, Masayuki Murata

**Affiliations:** ^1^Graduate School of Information Science and Technology, Osaka University, 1-5 Yamadaoka, Suita, Osaka 565-0871, Japan; ^2^Center for Information and Neural Networks (CiNet), National Institute of Information and Communications Technology (NICT) and Osaka University, 1-4 Yamadaoka, Suita, Osaka 565-0871, Japan

## Abstract

The concept of biologically inspired networking has been introduced to tackle unpredictable and unstable situations in computer networks, especially in wireless ad hoc networks where network conditions are continuously changing, resulting in the need of robustness and adaptability of control methods. Unfortunately, existing methods often rely heavily on the detailed knowledge of each network component and the preconfigured, that is, fine-tuned, parameters. In this paper, we utilize a new concept, called attractor perturbation (AP), which enables controlling the network performance using only end-to-end information. Based on AP, we propose a concurrent multipath traffic distribution method, which aims at lowering the average end-to-end delay by only adjusting the transmission rate on each path. We demonstrate through simulations that, by utilizing the attractor perturbation relationship, the proposed method achieves a lower average end-to-end delay compared to other methods which do not take fluctuations into account.

## 1. Introduction

In wireless ad hoc networks, it is known that transmissions over wireless channels suffer from radio propagation loss, shadowing, fading, radio interference, and limited bandwidth. Moreover, there are also effects from traffic patterns which can degrade certain links if the network control is not traffic aware. Therefore, a lot of research attempts have been made in every layer and even across layers to improve the performance of communications in ad hoc networks. However, most improvements consider only the existing problems and lack the flexibility towards emerging problems, especially the highly focused cross-layer optimization becomes less extensible and difficult to maintain [1].

Traditional network control mechanisms often rely on a certain set of predefined rules and fine-tuned parameters for known situations. However, computer network architectures and their protocols have become increasingly sophisticated over time through addition of many features to support new applications, where different applications may require different settings of protocol parameters. Since the total number of possible situations occurring in the real world is too numerous to be handled by preprogrammed sets of definitions, it is necessary that new networking mechanisms are designed in a flexible and adaptive manner to cater for any changes in the environment.

In an attempt to design new adaptive networking methods, concepts based on biological mechanisms have been proposed [2, 3] for self-organized control since they are able to provide greater robustness and adaptability to external influences. The core idea is to derive a protocol that is based on the model of a natural phenomenon. For example, swarm intelligence is a concept where individual agents mimic the behavior of foraging ants or bees in insect swarms and it has been successfully applied to routing problems in the past [4]. Firefly groups perform a distributed synchronization of their flashing behavior and this is applied to synchronization in sensor networks [5]. Reaction-diffusion describes the chemical dynamics of morphogens in the development of stripes or spots on animal furs. Based on the reaction-diffusion dynamics, the coding rate for camera sensor networks can be controlled [6].

Since biological systems are often described as dynamic systems, they rely on a mathematical formulation given as differential equations. In dynamic systems, attractors describe the states to which the system evolves over time. In the past, we studied the concept of attractor selection, which is based on the dynamics found in gene expression [7] and has been previously also applied to tackle problems in communication networks [8, 9]. In this paper, we apply a similar biological mechanism called attractor perturbation (AP), which is derived from the fluctuation-response relationship observed in an experiment on the evolution of functional proteins in a cell [10]. A previous application of AP to computer networks can be found in [11, 12].

In this paper, we focus on bandwidth improvement and end-to-end delay minimization in ad hoc networks. In terms of bandwidth improvement, one of the most common approaches is to use multiple paths in the same or across different media (multihoming). To enable the ability to utilize multiple paths concurrently, there is some existing work in both wired, for example, opportunistic multipath scheduling (OMS) [13], and wireless networks, for example, concurrent multipath transfer (CMT) [14, 15] and adaptive load balancing algorithm (ALBAM) [16]. However, most existing control methods require a full knowledge of the current network status, for example, queue length on each node, which is difficult to obtain or requires frequent probing causing bandwidth degradation. Therefore, we apply AP to concurrent multipath traffic distribution to improve the available bandwidth while utilizing the AP relationship to predict the outcome of the traffic adjustment and also minimize the end-to-end delay at the same time.

The contributions of this paper are as follows. First is the end-to-end characteristics of the AP-based proposal, which allows easy deployment in existing networks without the need of modifying all intermediate nodes. Second is the usage of statistical information, which consumes less bandwidth to obtain than using probing results. Third is the ability to provide a simplified view of the network as a black box with only the end-to-end observed variables while maintaining the ability to influence the network performance. Last is the expectable adaptability of the proposal since it does not rely only on the predefined parameters but also takes into account the fluctuation as a source of robustness and adaptability in a similar manner to other biological mechanisms.

The rest of this paper is organized as follows. We first explain the biologically inspired attractor perturbation model which is the basis of this study in Section 2. Next, we describe our proposal, that is, a concurrent multipath traffic distribution method, in Section 3. Then, the evaluation results from simulation are presented and discussed in Section 4. Finally, we conclude this paper and describe future work.

## 2. Attractor Perturbation

 The attractor perturbation model is derived from observations of fluctuation and response in biological systems, in particular, an experiment on the evolution of functional proteins in clone bacteria cells. 

### 2.1. Mathematical Model

 In [10], it was found that the fluctuation, which is expressed by the variance of the fluorescence of a bacterial protein, and its response, which is the average change in this fluorescence against the applied force, have a linear relationship modeled as follows:

(1) $$\begin{array}{c} {\overset{-}{x}}_{a+\Delta a}-{\overset{-}{x}}_{a}=b\Delta a\sigma_{a}^{2}, \end{array}$$

where *b* is a scalar constant, *x* is a time dependent measurable variable in the system with mean $\overset{-}{x}$ and variance *σ*_*a*_^2^, and *a* is the amount of force applied to the system. The attractor perturbation model is very similar to the fluctuation-dissipation theorem in physics.

There are two major assumptions underlying the model formulation of AP. First, the variable *x* must have a Gaussian-like distribution which is often observed in biology. Second, the variable *x* and the parameter *a* are closely associated. In other words, a change in the parameter *a* would strongly affect the distribution of the variable *x*.

### 2.2. Applications of AP Model

**Figure 1:**
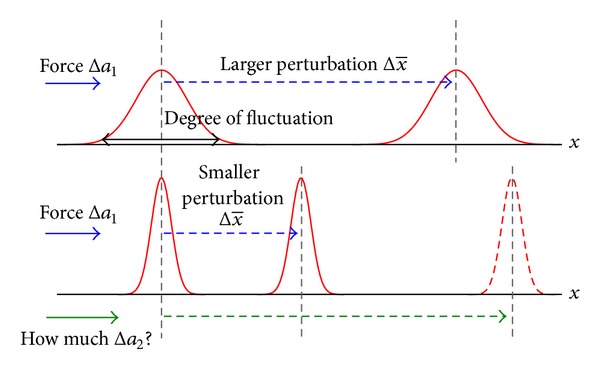
Dynamics of attractor perturbation.

 Equation (1) reveals that the difference in the average of the variable *x* before and after applying a change to the parameter *a* is linearly proportional to the amount of change in *a*, which is the force Δ*a*, and the variance of the variable *x* prior to the change. Therefore, one can predict the response to the applied force from the fluctuation of the targeted system. Since the amount of change in *a* can be seen as controllable, it is possible to adjust the difference in average of *x*, called *perturbation*, by taking the current variance of *x* into consideration. Obviously, using the same amount of force Δ*a* to perturb the average of *x* when the variance *σ*_*a*_^2^ is large will also lead to a larger perturbation, as shown in Figure 1. Based on this relationship, we use this model to estimate the amount of force required to achieve the desired amount of perturbation.

 To confirm the applicability of the AP model in our proposal, we first observed the delay distribution in ad hoc networks and discovered that it resembles a Gaussian distribution. Second, a related study has already shown the applicability of AP to traffic rate control for achieving a target delay in wired networks [12]. Since AP allows simplifying the underlying system as a black box by observing only the end-to-end variables, it is a promising way to reuse the concept of rate control in ad hoc networks. Hence, we decided to use AP for concurrent multipath traffic distribution by performing traffic rate control on each path to achieve overall higher bandwidth and lower end-to-end delay as explained in the next section.

## 3. Concurrent Multipath Traffic Distribution

 The advantage of using multiple paths is that if one path breaks due to failures at intermediate links or nodes, at least one other path can still be maintained. Furthermore, using multiple paths permits a better load balancing by distributing traffic more evenly in the network. Particularly, if nodes in an ad hoc scenario are operated by batteries, this may lead to reduced energy consumption of intermediate nodes. Finally, using multiple paths concurrently can improve the total available bandwidth in the network.

In today's wireless networks, it becomes common that participating devices can connect to more than one radio access technologies (RATs) and even within the same RAT, there are possibly multiple separated channels to use. Therefore, the concept of multipath can now be extended to multichannel and multihoming in heterogeneous wireless networks. Even though our current work focuses on ad hoc networks, our concept of path is still applicable to traffic allocation over multichannel and multihoming scenarios. The allocation granularity, which describes the unit of information allocated to each path, is also of great importance [17]. Coarse granularities, such as per connection or per flow, tend to reduce the management overhead but are not as flexible as small granularities, for example, per packet, since these permit a better distribution of traffic. However, per-packet granularity may require reordering at the destination, if the latencies differ too much among paths.

### 3.1. System Model

**Figure 2:**
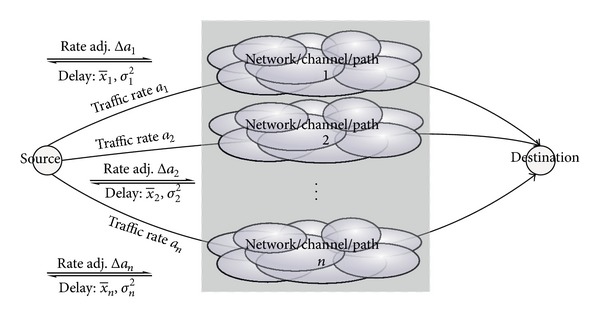
Overall system model.

In this study, we consider a situation where a source node is connected to the destination node via multiple paths and each path *i* does not cause interference with another as illustrated in Figure 2. This network model covers both ad hoc (or mesh) networks with multiple radio channels and also multihoming systems. For the sake of simplicity, we consider only *n* = 2 in this paper, but the proposed method can be easily extended to *n* > 2 cases as shown in the appendix.

The notations of variables on each path *i* are as follows:

- observed end-to-end delay (measurable variable): *x*_*i*_,
- current traffic rate (controllable variable or force): *a*_*i*_,
- amount of traffic rate adjustment: Δ*a*_*i*_,
- average end-to-end delay prior to applying Δ*a*_*i*_: $\overset{-}{x}{}_{i}$,
- average end-to-end delay after applying Δ*a*_*i*_: $\overset{-}{x}{}_{i}^{\prime }$,
- delivered packet count: *n*_*i*_.

### 3.2. Problem Definition

 Our proposal aims at minimizing the average end-to-end delay of all packets. Using AP, we attempt to minimize the *total delay sum*, which directly corresponds to the average delay of all packets on both paths. The delay sum can be estimated through the product of the expected delay and the adjusted traffic rate on each path.

According to the AP concept, in case of two paths, we have the expected average delay $\overset{-}{x}{}_{i}^{\prime }$:

(2) $$\begin{array}{c} {\overset{-}{x}}_{1}^{\prime }={\overset{-}{x}}_{1}+b_{1}\Delta a_{1}\sigma_{1}^{2},\\ {\overset{-}{x}}_{2}^{\prime }={\overset{-}{x}}_{2}+b_{2}\Delta a_{2}\sigma_{2}^{2}. \end{array}$$

Therefore, we can define a function *f*(Δ*a*_1_, Δ*a*_2_) as an estimation of the average delay after applying traffic rate adjustment Δ*a*_*i*_ as follows:

(3) f(Δa1,Δa2)  =(a1+Δa1)x−1′+(a2+Δa2)x−2′  =(a1x−1+a2x−2)+(x−1+a1b1σ12)Δa1   +(x−2+a2b2σ22)Δa2+b1σ12Δa12+b2σ22Δa22.

Given that $c^{\prime }=\left(a_{1}{\overset{-}{x}}_{1}+a_{2}{\overset{-}{x}}_{2}\right)$, $c_{1}=\left({\overset{-}{x}}_{1}+a_{1}b_{1}\sigma_{1}^{2}\right)$, $c_{2}=\left({\overset{-}{x}}_{2}+a_{2}b_{2}\sigma_{2}^{2}\right)$, *k*_1_ = *b*_1_ *σ*_1_^2^, and *k*_2_ = *b*_2_ *σ*_2_^2^, (3) can be formulated as a constrained optimization (minimization) problem as follows:

(4) $$\begin{array}{c} \begin{array}{cc} \text{Minimize} & f\left(\Delta a_{1},\Delta a_{2}\right)=c^{\prime }+c_{1}\Delta a_{1}+c_{2}\Delta a_{2}\\ & +k_{1}\Delta a_{1}^{2}+k_{2}\Delta a_{2}^{2}\\ \text{subject to} & \Delta a_{1}+\Delta a_{2}=0. \end{array} \end{array}$$

The solution of the minimization problem in (4) is the amount of the adjustment in traffic rate to be applied to each path in order to achieve minimal average end-to-end delay of all packets. The *subject to* condition is required since the total amount of traffic prior to and after the adjustment has to remain the same.

### 3.3. Lagrangian Optimization

 The minimization problem which has the form as in (4) can be solved using *Lagrangian Optimization*.

The Lagrangian has the general form of

(5) $$\begin{array}{c} L\left(x^{*},\lambda^{*}\right)=f\left(x\right)-\Sigma_{i}\left[\lambda_{i}\left(g_{i}\left(x\right)-b_{i}\right)\right], \end{array}$$

where x* is the optimal solution of *x* and λ* is the penalizing Lagrangian multiplier.

The associated Lagrangian of (4) is:

(6) L(Δa1∗,Δa2∗,λ∗)=c′+c1Δa1∗+c2Δa2∗+k1Δa1∗2 +k2Δa2∗2−λ∗(Δa1∗+Δa2∗),

(7) $$\begin{array}{c} \frac{\partial L}{\partial \Delta a_{1}^{*}}=c_{1}+2k_{1}\Delta a_{1}^{*}-\lambda =0, \end{array}$$

(8) $$\begin{array}{c} \frac{\partial L}{\partial \Delta a_{2}^{*}}=c_{2}+2k_{2}\Delta a_{2}^{*}-\lambda =0, \end{array}$$

(9) $$\begin{array}{c} \frac{\partial L}{\partial \lambda^{*}}=-\left(\Delta a_{1}^{*}+\Delta a_{2}^{*}\right)=0. \end{array}$$

In the three equations (6)–(8), there are three unknown variables Δ*a*_1_*, Δ*a*_2_*, and λ*. Therefore, this optimization problem can be solved and we obtain the optimal amount of traffic rate adjustment Δ*a*_*i*_ for each path *i* to minimize the sum of average delays.

According to the steps taken previously, the optimal solution in case of two paths is as follows:

(10) Δa1∗=c2−c12(k1+k2)=(x−2+a2b2σ22)−(x−1+a1b1σ12)2(b1σ12+b2σ22),Δa2∗=−Δa1∗.

### 3.4. Traffic Distributing Steps

The optimal solution Δ*a*_i_* from (10) is used in Algorithm 1, executed at the source every interval of duration *ρ* (=5 s in our simulation experiments).

In every iteration of the algorithm, our main AP-based traffic control method (AP−Com) uses the measured average $\overset{-}{x}{}_{i}$, the variance *σ*_*i*_^2^, and the current traffic rate *a*_*i*_ to solve the minimization problem. The optimal solution is applied to the current traffic rate gradually, which is limited by the maximum rate adjustment ratio *α*_max⁡_.

 In addition to our main proposal AP−Com, a variation of AP-based method with delay compensation (AP+Com) is also proposed here. The delay compensation process serves to maintain throughput in our mechanism at the cost of using more information of the delivered packet count *n*_*i*_ on each path *i* for calculating the number of lost packets. Without delay compensation, AP−Com behaves in favor of lower delay regardless of the delivery performance on each path. In AP+Com, we compensate for packet loss assuming that each lost packet has the end-to-end delay equal to *ρ*. We will show results of both AP-based methods with and without delay compensation in the evaluation section.

The coefficient *b*_*i*_ is required to solve the minimization problem. This value is crucial for estimating the average delay after applying the traffic rate adjustment and is determined in every iteration based on the current iteration's average delay, the previous iteration's average delay and variance, and the amount of rate adjustment applied in the previous iteration using (1). In case that there is no traffic rate adjustment in the previous iteration, the default *b* given in the configuration is used in that iteration and the accurate *b*_*i*_ can be calculated on the subsequent iteration. Note that another study on AP in wired networks [12] has found that the AP-based control does not require a fine tuning of coefficient *b*. We will show later in Section 4.4 that the same concept also holds in our wireless case.

## 4. Evaluation

To demonstrate the validity of the AP-based traffic distribution, we performed simulations using the QualNet network simulator. We divided the evaluation into two parts, throughput in a static scenario and end-to-end delay improvement in mobile scenarios.

### 4.1. Comparison Target

 In the static scenario evaluation, we compare the performance of the AP-based proposal to two existing multipath transport layer control protocols: concurrent multipath transfer (CMT) [14] and multipath real-time transport protocol (MPRTP) [18]. CMT utilizes the SCTP [19] protocol to send data concurrently to the destination, which has multiple networking interfaces, while MPRTP is an extended version of the RTP protocol to allow scheduling of RTP traffic over multiple paths concurrently.

 Our proposal is similar to the recently proposed MPRTP protocol because both are implemented over UDP. The scheduling algorithm of MPRTP uses loss rate, packet sizes, bytes sent, and interval information from RTP's receiver reports (RRs) to estimate the bandwidth of the current path. Using packet loss information, paths are categorized as congested, mildly congested, and non-congested conditions. The scheduler then continuously assigns a portion of traffic to each path, more if it is noncongested and less if it is congested, while keeping the same total rate. We have implemented Algorithm 1 of MPRTP from [18] in QualNet, assuming a perfect knowledge of end-to-end information instead of using real RR packets, and compare its performance with our proposal.

As already mentioned previously, CMT is implemented over SCTP, which is supported by the IETF alongside with TCP and UDP as a general purpose reliable transport protocol with connection-oriented, reliable data transfer, window-based, congestion control, and flow control features, similar to TCP. One important feature of SCTP is its built-in multihoming capability where a connection can be established between a set of IP addresses. However, standard SCTP uses only a pair of primary IP addresses at a time which does not allow concurrent transmissions. CMT is a modified version of SCTP that allows concurrent transmissions and includes few improvements on fast retransmission, congestion window update, and delayed acknowledgment algorithms. It was found in [15] that the receiving buffer, referred to as *rBuf* in the original paper, can be a performance bottleneck of CMT. Therefore, we only compare the best CMT results without such constraint, called *CMT Unlimited*, as a reference in this section.

 We did not implement our proposal over TCP or SCTP because in the TCP scheme, due to various control mechanisms, for example, rate control and congestion avoidance control, end-to-end delays do not generally follow a Gaussian distribution. There are a few special cases when TCP traffic does follow a Gaussian distribution [20, 21]; however, we leave the investigation of those cases as a future work.

 Currently, to study the pure behavior of our proposal, we assume that the end-to-end information is known to the source node without an actual measurement. However, a feedback mechanism can be easily implemented to deliver this information to the source node. Since the statistical information is needed only once every execution interval, the overhead can be considered negligible and the actual results should be similar to the simulation results shown in this paper. Moreover, to have a fair comparison, our comparison targeting MPRTP also uses the same assumptions.

### 4.2. Static Scenario

**Figure 3:**
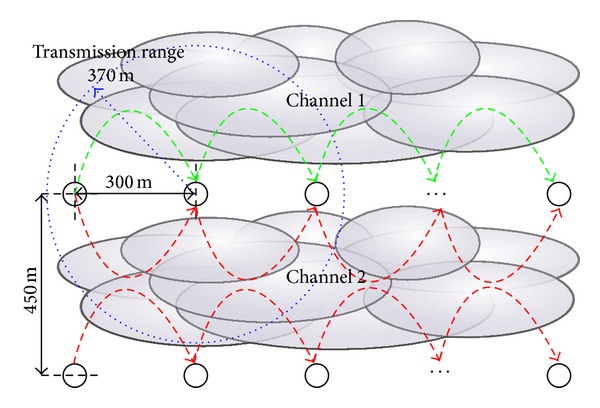
Simulation scenario from [15].

We set up the simulation scenario exactly the same as described in [15]; see Figure 3. There are two chains of nodes where the distance between nodes on the same chain is 300 m and the distance between chains is 450 m. The transmission range of each node is approximately 370 m where the carrier sensing range and the interference range span farther under the two-ray path loss model without fading. The default transmission range in QualNet 5.2 is only 300 m and we matched the transmission range to [15] by slightly increasing the TX power.

In this scenario, one chain serves as the main concurrent multipath sessions for bandwidth evaluation and the other chain serves as interfering background traffic. On the main chain, each node is equipped with two IEEE 802.11b interfaces connected to two noninterfering channels. On the background traffic chain, each node is equipped with only one interface connected to the second channel which is used in the main chain. The data rate for IEEE 802.11b is 2 Mbps and the RTS/CTS mechanism is enabled. Static routes are used in this simulation to eliminate complications due to the effect of the routing protocol.

**Figure 4:**
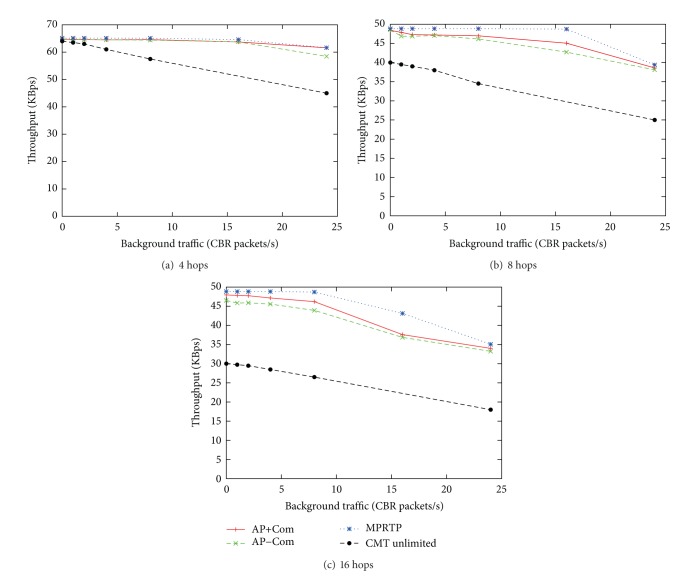
Throughput comparison between AP-based proposals, MPRTP, and CMT unlimited *rBuf* from [15].

The number of nodes varies from 10 to 34 (4, 8, and 16 hops on each chain). The traffic used in this evaluation is CBR with 1000 bytes per packet. We performed the simulations using several different traffic rates on the main chain and have selected the one with the highest obtained throughput shown in Figure 4. The main total traffic rates for the 4, 8, and 16 hop cases are 65.1, 48.8, and 48.8 KBps, respectively, which are decided based on the number of hops to the destination and the ratio of capacity explained in [22]. The main traffic is sent from the source during 60–360 seconds in a 420-second long simulation. The amount of background traffic varies from 0 to 24 packets per second. The results of our protocol shown in Figure 4 are the average of 30 runs, which is the same number of runs performed in [15].

From Figure 4, it can be clearly seen that in comparison to CMT, MPRTP, and AP-based methods (with *b* = 0.1 and *α*_max⁡_ = 0.1) can achieve much higher throughput and are less susceptible to the interference from background traffic. Even though now the implementations of both AP and MPRTP methods do not fully use feedback packets to gather the statistical information, a single feedback packet per decision interval *ρ* (=5 s in this study) can hardly affect the higher bandwidth shown here. Therefore, we can claim here that the AP-based method and MPRTP are viable alternatives to CMT, which can provide better bandwidth improvement when an application can tolerate or handle packet loss.

 Among UDP-based proposals, MPRTP could achieve higher bandwidth due to its accurate rule-based bandwidth prediction in cases of low interference and background traffic load. However, when congestion occurs and more packet loss is observed, the bandwidth difference becomes smaller. Since MPRTP relies heavily on the information accuracy, the smaller difference is most likely due to the lower accuracy of rule-based bandwidth prediction of MPRTP.

 A similar behavior can be observed between AP+Com, which estimates delay compensation using packet loss, and AP−Com, which does not use delay compensation. With the delay compensation process added in AP+Com, the performance of the AP-based method is slightly better than in AP−Com because the compensated delay reflects the actual network conditions better and enhances the accuracy of AP in estimating delay after adjusting the traffic rate. However, the performance difference becomes smaller in the same manner to MPRTP when the load is high.

 It is important to emphasize that while using much less information, that is, only delay information without delivered packet count nor lost packet count in comparison to AP+Com and MPRTP, AP−Com can achieve comparable throughput to other protocols. This is a piece of evidence of the adaptability of the AP-based methods, which uses delay fluctuations, and further supportive results will be shown in the next subsection.

### 4.3. Mobile Scenario

 In this section, we evaluate the average delay of the AP-based proposals with *b* = 0.1 and *α*_max⁡_ = 0.1 in mobile scenarios. In such scenarios, an adaptive traffic distribution method is required since a traffic pattern on a certain path is affected by changes in other paths due to rerouting, topology changes, and so forth. Most concurrent multipath traffic distribution methods do not support/consider mobile scenarios. Therefore, in addition to the baseline strategy where the traffic is split evenly on both paths (*evenly distributed*) and the MPRTP approach, we developed another comparison method, called *heuristic* method, which operates based on the end-to-end average delay in a similar manner to our AP-based method. The main differences are that the heuristic method

- adjusts the traffic with the fixed ratio of the total traffic rate *α*_max⁡_ = 0.1 (the AP-based method calculates the optimal solution in the range of [−*α*_max⁡_, *α*_max⁡_]),
- cannot estimate the delay after applying the traffic rate adjustment, and
- makes the decision to transfer the traffic purely from the path with higher average delay or the path with higher loss rate (in case of no delivered packet) to the path with lower one.

We expect that the evaluation against the *heuristic* method will reveal the importance of taking the fluctuation into account when performing traffic distribution.

The scenario settings are as follows. 100 mobile nodes are distributed randomly in a 1500 × 1500 m^2^ area. The random waypoint model is used with a minimum speed of 2 m/s, a maximum speed of 10 m/s, and a pause time of 30 s. Each node is equipped with two 802.11b interfaces with the data rate of 2 Mbps, connected to two noninterfering radio channels. There is one main multipath traffic session with total traffic rate of 20 packets/s and the packet size of 1000 bytes, which is the same as the previous scenario. The number of background CBR traffic sessions varies from 0, 4, 8, and 12 to 16 sessions per channel. Every background traffic session has the traffic rate of 1 packet/s. We chose a relatively low bit rate of background traffic to only increase interference, while ensuring sufficient bandwidth for the main session to avoid overloading conditions, in which we cannot evaluate the performance of traffic distribution methods.

**Figure 5:**
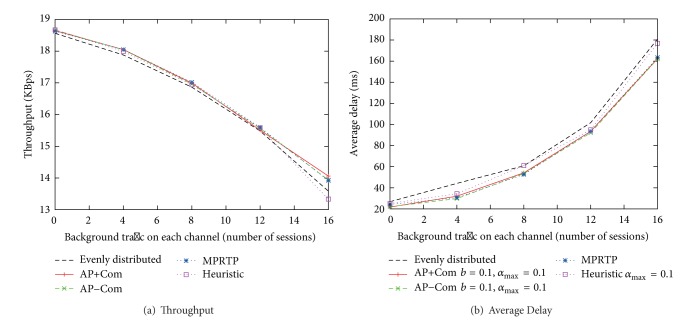
Performance comparison under mobility scenario.

**Figure 6:**
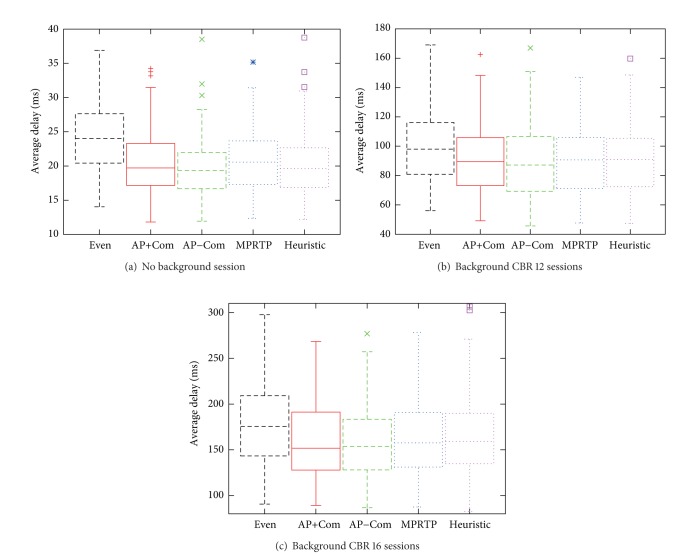
Average delay comparison under mobility scenario (*y*-axis is capped for visibility).

 The average results from 100 runs are shown in Figure 5. Figure 5 shows the throughput and average delay against the amount of background traffic. Since the differences between each curve in Figure 5(b) cannot be clearly seen, more details of average delay on each run is shown in Figure 6 using box-and-whisker diagram where the box reflects the lower quartile (Q1), median (Q2), and upper quartile (Q3). The bars show the range of ±1.5 IQR and the dots show the data that are outside the range.

 It can be observed from Figure 5 that the throughput of each approach is quite similar. However, there is a difference in average delay as shown in Figure 5(b). It is out of question that the baseline approach without traffic redistribution (*evenly distributed*) has the worst average delay. Our AP proposals can achieve the same level of average delay as MPRTP by using only end-to-end delay statistics. The newly proposed comparison method (*heuristic*), which uses only average end-to-end delay, performs much worse than the AP proposals because using only the average delay cannot provide a good estimate of the path quality, that is, congestion level.

Moreover, Figure 6 indicates that the median of all methods generally follow the same tendency of the average, except the heuristic one. This is an effect from cases where the average delay is very high (capped and cannot be seen in the figure). Those cases are caused by the inappropriate traffic distribution that induced high congestion, which consequently causes failure in routing, hence, a much higher end-to-end delay.

According to these results, it can be understood that AP-based methods, which use both average and variance, can perform better than methods using only the average, like heuristic. Therefore, it is safe to claim that considering not only the average delay in the current interval, but also the fluctuation is important for improving the performance of the traffic distribution method.

 Additionally, by using only the statistical information on delay, AP−Com can achieve comparable throughput and end-to-end delay to MPRTP, which requires more information of delivered bytes and loss rate. Hence, it is confirmed that the AP-based method does not need the details of the system under its control, which is preferable from an implementation viewpoint because a high processing overhead, energy consumption, and errors from actual measurements can be avoided.

### 4.4. Discussion on Bio-Inspired Adaptability

 From Figures 5 and 6, it can be seen that AP−Com is the best among all approaches. Even though the throughput results of AP−Com in the static ad hoc network scenario were slightly lower than the other approaches, it can adapt well to scenarios with higher dynamics. This result conforms with our previous assumption regarding the rule-based bandwidth prediction of MPRTP and the delay compensation of AP+Com and shows that a bio-inspired method indeed reveals better adaptability to different scenarios without the need of fine-tuning parameters. 

**Figure 7:**
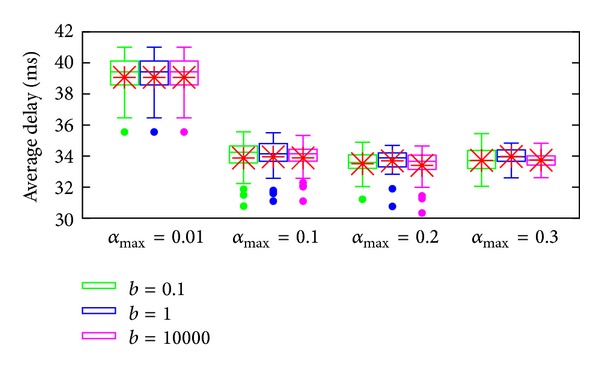
Results of AP+Com with different values of *b*.

To further support this claim, we also added the results from bandwidth improvement scenario with different coefficients *b* in Figure 7. It can also be seen that even with inaccurate *b* for a specific scenario, the AP-based method can adapt to that situation and perform considerably well, due to its core bio-inspired model.

## 5. Conclusion

We presented a novel biologically inspired concurrent multipath traffic distribution method based on attractor perturbation. In AP, the whole underlying system is regarded as a black box and its control is based on the observed average and variance of the time series of the considered performance metric. Therefore, our proposal requires only end-to-end statistical information to perform traffic distribution. From simulation results, we have shown that our main proposal (AP−Com) can achieve lower average end-to-end delay without sacrificing throughput when compared to the *heuristic* method and evenly distributed traffic on all paths. Moreover, it can even achieve similar average end-to-end delay as MPRTP, which uses delivered bytes and loss rate in addition to delay information. It is natural that a mechanism using more information achieves better performance, but it suffers from inaccuracy of obtained information and also requires parameters fine tuning. An evaluation of cases with information errors remains future work.

In addition to the performance aspect, our proposal does not require any careful parameter fine tuning due to its bio-inspired nature. The usage of fluctuation, or noise, within the core AP model gives it a flexibility to handle frequent changes in the network. It is also expected that with this adaptability, our proposal should be able to handle emerging problems better than traditional methods.

## Figures and Tables

**Algorithm 1:**
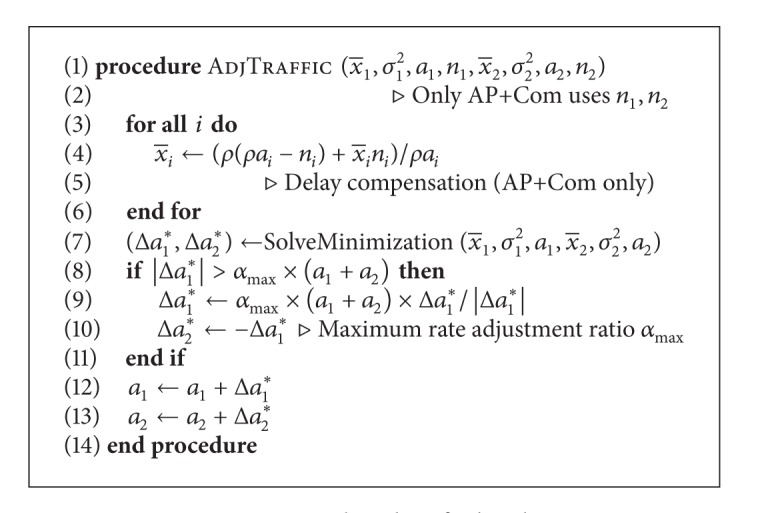
AP-based traffic distribution.

## Appendix

### Minimization Problem: *n*-Path Case

According to the AP concept, in case of *n* paths, we have

(A.1) x−1′=x−1+b1Δa1σ12⋮ x−n′=x−n+bnΔanσn2.

Total delay sum of *n*-path case can be calculated as follows:

(A.2) f(Δa1,Δa2,…,Δan) =(a1+Δa1)x−1′+⋯+(an+Δan)x−n′ =(a1+Δa1)(x−1+b1Δa1σ12)+⋯  +(an+Δan)(x−n+bnΔanσn2) =(a1x−1+⋯+anx−n)+(x−1+a1b1σ12)Δa1+⋯  +(x−n+anbnσn2)Δan+b1σ12Δa12+⋯+bnσn2Δan2 =Σin(aix−i+(x−i+aibiσi2)Δai+(biσi2)Δai2).

The minimization problem can be formulated similarly to the 2-path case

(A.3) $$\begin{array}{c} \begin{array}{cc} \text{Minimize} & f\left(\Delta a_{1},\Delta a_{2},\ldots ,\Delta a_{n}\right)\\ \text{subject to} & \Sigma_{i}^{n}\Delta a_{i}=0. \end{array} \end{array}$$

The associated Lagrangian of (A.3) is

(A.4) L(Δa1∗,…,Δan∗,λ∗)=Σin(aix−i+(x−i+aibiσi2)Δai∗+(biσi2)Δai∗2) −λ∗ΣinΔai∗,∂L∂Δa1∗=a1b1σ12+2b1σ12Δa1∗−λ=0⋮ ∂L∂Δan∗=anbnσn2+2bnσn2Δan∗−λ=0,∂L∂λ∗=−ΣinΔai∗=0.

From (A.4), we can form an augmented matrix as follows:

(A.5) $$\begin{array}{c} \left[\begin{array}{ccccccc} 2b_{1}\sigma_{1}^{2} & 0 & 0 & \cdots & 0 & -1 & -2b_{1}\sigma_{1}^{2}\\ 0 & 2b_{2}\sigma_{2}^{2} & 0 & \cdots & 0 & -1 & -2b_{2}\sigma_{2}^{2}\\ \vdots & \vdots & \ddots & \cdots & \vdots & \vdots & \vdots \\ 0 & 0 & \cdots & 2b_{n-1}\sigma_{n-1}^{2} & 0 & -1 & -2b_{n-1}\sigma_{n-1}^{2}\\ 0 & 0 & \cdots & 0 & 2b_{n}\sigma_{n}^{2} & -1 & -2b_{n}\sigma_{n}^{2}\\ 1 & 1 & \cdots & 1 & 1 & 0 & 0 \end{array}\right]. \end{array}$$

This augmented matrix can be solved using row elimination.
